# Suitable Heel Height, a Potential Method for Musculoskeletal Problems during the Third Trimester: A Pilot Study

**DOI:** 10.3390/bioengineering11070667

**Published:** 2024-06-29

**Authors:** Linjuan Wei, Yan Wang, Yinghu Peng, Guoxin Zhang, Qitao Tan, Yaodong Gu, Ming Zhang

**Affiliations:** 1 Department of Biomedical Engineering, Faculty of Engineering, The Hong Kong Polytechnic University, Hong Kong 999077, China; linjuan-jo.wei@connect.polyu.hk (L.W.); guo-xin.zhang@connect.polyu.hk (G.Z.); matthew.tan@connect.polyu.hk (Q.T.); 2 Shenzhen Research Institute, The Hong Kong Polytechnic University, Shenzhen 518057, China; 3 Research Institute for Sports Science and Technology, The Hong Kong Polytechnic University, Hong Kong 999077, China; 4 CAS Key Laboratory of Human-Machine Intelligence-Synergy Systems, Shenzhen Institutes of Advanced Technology Chinese Academy of Sciences, Shenzhen 518055, China; yh.peng@siat.ac.cn; 5 Faculty of Sports Science, Ningbo University, Ningbo 315211, China; guyaodong@nbu.edu.cn

**Keywords:** third trimester, heel height, gait biomechanics

## Abstract

Background: The treatment options for third-trimester musculoskeletal issues are limited. This study aims to examine how heel height affects gait biomechanics and provides heel height recommendations for various musculoskeletal problems. Methods: Five third-trimester gravidas were recruited wearing uniform footwear with four heel heights (0 mm, 15 mm, 30 mm, and 45 mm). Lower-limb muscle forces, joint angles, joint torques, joint contact forces, and ground reaction forces (GRF) at specific moments (the first peak, valley, and second peak of GRF) were collected for one-way analysis of variance with repeated measures. Results: The soleus, gastrocnemius, tibialis posterior, plantaris, obturator externus, gluteus maximus, gemellus superior, and obturator internus were the smallest at heel heights of 45 mm and 15 mm at the valley of GRF. Hip extension and knee flexion displayed the smallest joint angle and joint torques at a height of 15 mm. Ankle joint contact force decreased with increased heel height. Conclusions: The height of the heel significantly impacts muscle force, joint angles, joint torques, and joint contact force. A heel of 15 mm might be the most suitable heel height to potentially avoid or alleviate musculoskeletal problems during the third trimester.

## 1. Introduction

Throughout pregnancy, women experience numerous physical and hormonal changes. The fluctuations of hormones, particularly the increased levels of estrogen, progesterone, and relaxin, can lead to an increase in ligament laxity [1,2,3]. The heightened ligament laxity causes joints to become more flexible, leading to misalignment and higher strain [4]. The redistribution of weight caused by the fetus’s growth and repositioning, particularly in the third trimester (week 27 to the end of the pregnancy) [5,6], leads to misaligned lower-limb joint angles, decreased stability, and increased lower-limb loading [7]. These physical and biomechanical changes can result in musculoskeletal issues such as soft tissue swelling, lower back and lower-limb joint pain, carpal tunnel syndrome, and DeQuervain’s Tenosynovitis [8]. Additionally, the physiological adaptations within the body during pregnancy can significantly influence the utilization of all muscular systems, potentially resulting in muscular fatigue and subsequent pathological alterations [9].

Musculoskeletal complications during pregnancy are highly prevalent [10]. Possible reasons include a significant change in foot shape and size, decreased arch height, and increased foot length and width during pregnancy, especially from the second to third trimesters [11]. Additionally, body mass increases significantly in the third trimester, and loading is primarily concentrated on the heel during walking. Gravidas also causes a decrease in balance ability and the right ankle plantarflexion muscles are preferentially used to control movement [12,13]. Changes in foot dimensions may indicate the required shoe size and arch support need to be adjusted [14]. Improper shoes, including loose, backless, slippery, and high-heeled shoes (at least one inch), have been linked to falls during pregnancy [15,16]. Therefore, the study of shoes’ parameters for pregnant women in the third trimester to relieve foot-related discomfort and imbalance is necessary.

Heel height, a crucial parameter in the design of shoes, has long been a contentious topic in women’s health, as it can alter foot and ankle biomechanics [17,18]. Heel height has been demonstrated to have a relationship with the activity of muscles and the control of ankle joints [19,20]. As heel height increases, the pressure of the forefoot shifts toward the medial side [15]. If the heel height exceeds a certain threshold, it can increase the front ball of the foot’s load and strain the Achilles tendon and calf muscles, resulting in the alteration of balance, foot discomfort, and even foot deformities [21]. Although plantar pressure distribution, lower-limb joint force, torque, and muscle usage can be affected by heel height, the effects are not consistent in terms of biomechanics and musculoskeletal disorders, for example, in the influence of heel height on lower back pain. One study about heel heights of 5–7 cm showed that lower back pain is not associated with high heel height but is related to work years [22], but another study about heel height showed that increased heel height can lead to a decreased pelvic inclination angle, causing anterior tilt of the pelvis [23], which has a risk of non-specific chronic lower back pain [24].

This study aimed to explore the effect of heel height (0 cm, 1.5 cm, 3 cm, 4.5 cm) on the lower-limb biomechanics of pregnant women in the third trimester during walking. It was assumed that different heel heights would significantly change the muscle force, joint angles, joint torques, and joint contact force.

## 2. Materials and Methods

### 2.1. Participants

Five pregnant participants (Table 1) were recruited for this study. All of them had a dominant right limb, as determined by the test for dominant limb determination [25]. The inclusion criteria were: (1) healthy pregnant women; (2) third trimester (at least the 27th week); (3) physically active; and (4) no potential diseases that could influence walking ability. The exclusion criteria were: (1) neuromuscular disease; (2) biomechanical anomalies and problems impacting walking ability and performance; and (3) other diseases that are not suitable for a test, like high blood pressure. Written informed consent was obtained from each participant after introducing the test requirements and procedures. This study was performed in compliance with the declaration of Helsinki and approved by the Hong Kong Polytechnic University Human Subjects Ethics Sub-Committee (Reference No: HSEARS20210125008).

### 2.2. Procedures

**Figure 1:**
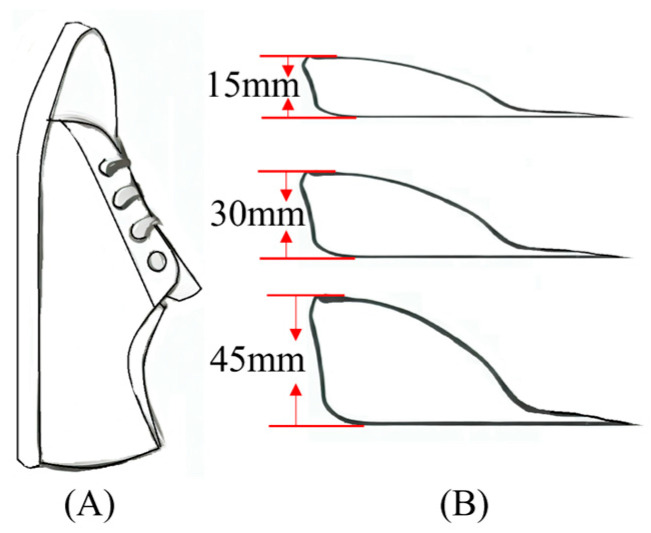
Schematic diagram of the shoe (**A**) and heel pads (**B**).

**Figure 2:**
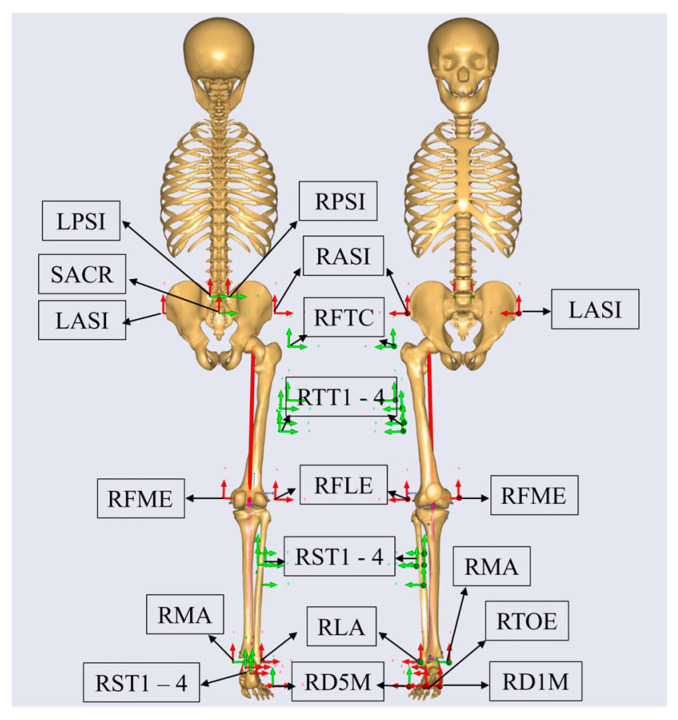
Location and names of the reflective markers. The black arrows direct to the marker labels. The arrows of other colors represent the direction of movement of the markers. Abbreviation: LASI/RASI = left/right anterior superior iliac crest; LPSI/RPSI = left/right posterior superior iliac crest; SACR = medial of the LPSI and RPSI; RFTC = right greater trochanter; RTT1—4 = four markers at the lateral side of 1/2 of the right thigh; RFLE = lateral epicondyle of the right femur; RFME = medial epicondyle of the right femur; RST1—4 = four markers at the lateral side of 1/2 of the right shank; RLA = right lateral ankle; RMA = right medial ankle; RHT1—3 = three markers located at the right heel; RD5M = distal lateral of the right fifth metatarsal; RD1M = distal medial of the right first metatarsal; RTOE = right head of the second metatarsal.

Before conducting the motion-capture walking, participants were asked to perform a five-minute warm-up walk and become familiar with the experiment environment. Gait analysis was systematically conducted on every participant under four conditions: walking while wearing shoes without a heel pad, and walking while wearing shoes with different heel pad heights (15 mm, 30 mm, 45 mm). All participants wore identical canvas shoes, and the heel pads utilized silicon material covered with fabric (Figure 1). All participants walked at their individual preferred speed through a 15-m track. A motion capture system (Vicon Motion System, Oxford Metrics Ltd., Oxford, UK) with eight infrared cameras and a force platform (OR6, AMTI, Watertown, USA) was used to collect gait data [26]. Detailed information about the chosen marker locations is shown in Figure 2. Participants continued to walk until five successful trials with the complete right stance phase on the platform were collected. There was a two-minute rest interval for each trial to avoid fatigue. The maker trajectories and vertical ground reaction forces (GRFs) were collected synchronously during the static and walking trials at sampling rates of 250 Hz and 2000 Hz, respectively.

### 2.3. Musculoskeletal Model Construction

Commercial musculoskeletal modeling software (AnyBody 6.0, Anybody Technology, Aalborg, Denmark) was used to estimate both lower-limb kinematics and kinetics [27]. The musculoskeletal (MSK) model, derived from the anthropometric database of the Twente Lower Extremity Model (TLEM 1.1) [28], facilitated the computation of joint movements and forces within the lower extremities. This comprehensive model includes segments such as the head, trunk, pelvis, femur, tibia, foot, and 57 muscle actuators [28]. The musculoskeletal multibody model calculation process was divided into three steps, namely, static scaling, inverse kinematics, and inverse dynamics. In the static scaling phase, the model utilized static experiment markers to scale bone lengths and optimize virtual markers within the musculoskeletal model. Additionally, we employed a length–mass–fat scaling approach to appropriately scale muscle strength within the generic musculoskeletal model [29]. Subsequently, motion capture data from walking trials was utilized to calculate joint kinematics parameters during the inverse kinematics step [30]. Finally, the calculated lower-limb kinematics and measured ground reaction forces were adopted as inputs to execute the inverse dynamic step, which estimated the lower-limb joint contact forces and muscle forces. More detailed information was reported in our previous study [31].

### 2.4. Data Processing and Statistical Analysis

**Figure 3:**
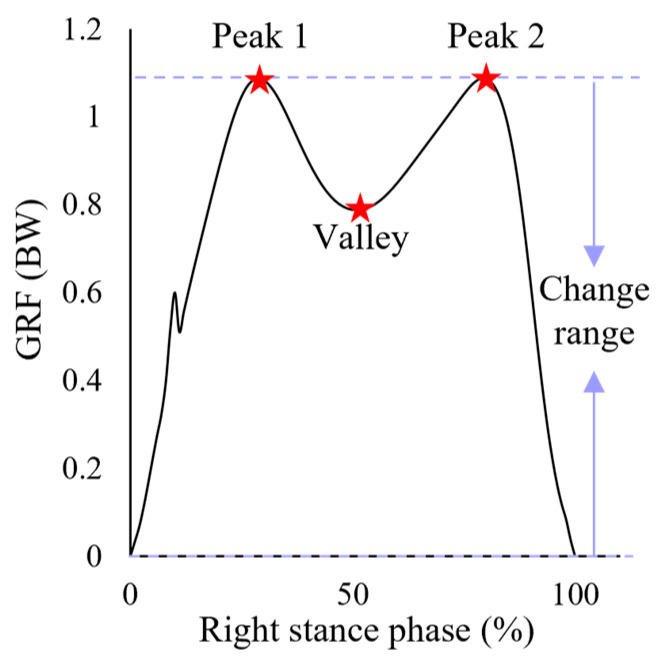
Illustration of peak 1, peak 2, the valley, and the change range.

The outcome variables included the vertical GRF, joint angles, joint torques, joint forces, and muscle forces during the stance phase. The data used in the further analysis were the values of those variables at the locations of peak 1 (P1), peak 2 (P2), and the valley (V0) of the vertical GRF, as well as the change ranges (range) of those variables (Figure 3). among four heel heights. P1 was defined as the highest value of the first 50% of the stance phase of the vertical GRF, which represents the maximum force experienced during the loading response phase. P2 was defined as the highest value of the second 50% of the vertical GRF, which is the maximum force during the propulsion phase. V0 was defined as the lowest value between P1 and P2, which represents the minimum force during the transition from the loading phase to the propulsion phase. The change range was defined as the highest value minus the lowest value. The analyzed joints included the hip, knee, and ankle of the right lower limb, and the analyzed muscles were the muscles around these three joints.

Muscle forces included soleus, gastrocnemius, flexor digitorum longus, flexor hallucis longus, tibialis posterior, tibialis anterior, peroneus brevis, peroneus longus, peroneus tertius, extensor digitorum longus, extensor hallucis longus, vastus lateralis, vastus medialis, vastus intermedius, rectus femoris, semitendinosus, semimembranosus, biceps femoris caput longum, biceps femoris caput breve, sartorius, iliacus, gluteus minimus, gluteus medius, gluteus maximus, tensor fasciae latae, piriformis, gracilis, adductor magnus, adductor brevis, gemellus inferior, gemellus superior, obturator externus, obturator internus, pectineus, plantaris, quadratus femoris, and psoas major.

The outcome variables from the musculoskeletal model analysis were then interpolated to 101 points for each trial. Following the interpolation, the next step involved averaging the stance phase data across the five trials for each participant. The analysis further extended to identifying key metrics at specific points during the stance phase: P1, P2, and V0 of the vertical GRF. At these points, the study examined the joint angles, joint torques, joint forces, and muscle forces, providing a comprehensive view of the biomechanical processes. Additionally, the range of changes in these variables was calculated to understand the variability among the five participants. The data were processed using MATLAB (version 2024a, The MathWorks Inc., Natick, MA, USA).

The joint contact forces and muscle forces were normalized to body weight (BW). The joint torques were normalized to body weight times body height (BW·BH). One-way analysis of variance (ANOVA) with repeated measures was conducted among four heel heights for each parameter at a significance level of 0.05 using SPSS (version 27.0, IBM, Chicago, IL, USA). If significant differences were found, the post-hoc pairwise comparison using Bonferroni correction was conducted.

## 3. Results

Table 2 presents the items that showed statistical significance in the analysis.

We conducted a comparison of the variables for different heel heights between 0 mm and 15 mm, 0 mm and 30 mm, 0 mm and 45 mm, 15 mm and 30 mm, 15 mm and 45 mm, and 30 mm and 45 mm. The greatest muscle force difference was displayed between heel heights of 0 mm and 15 mm, followed by 0 mm compared with 45 mm. For the joint angle, the most significant difference was observed between heel heights of 15 mm and 45 mm, followed by 30 mm and 45 mm. For the joint torque, the most significant difference was between 0 mm and 45 mm. The most significant difference for the ankle joint force was between 15 mm and 45 mm, followed by between 0 mm and 45 mm.

### 3.1. Muscle Force

**Figure 4:**
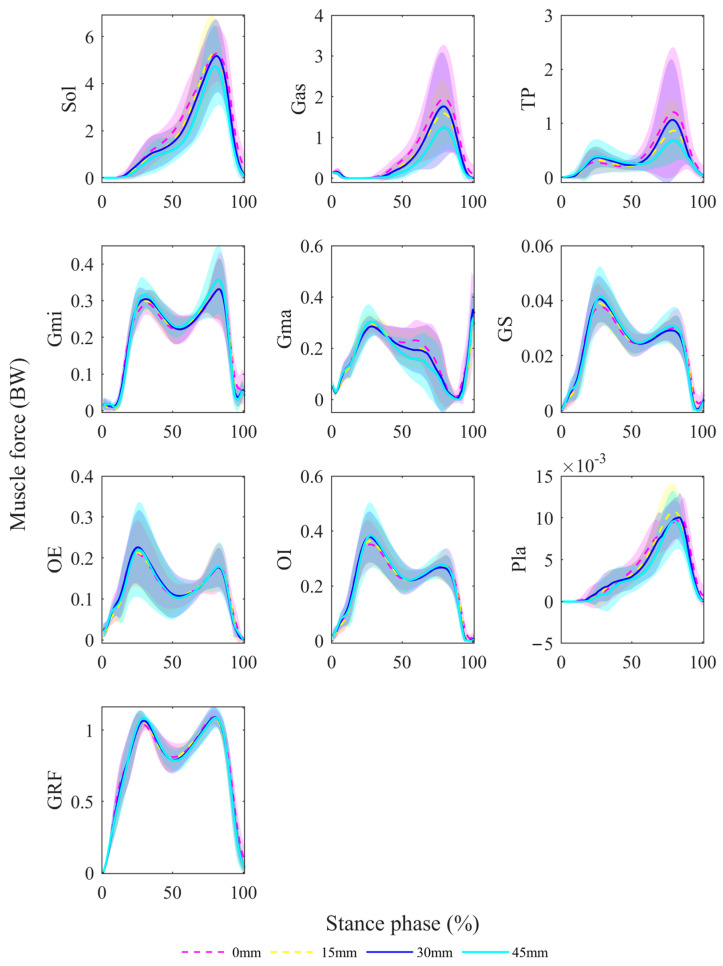
Comparisons of lower-limb muscle force and GRF during the stance phase under different shoe conditions. Sol: soleus muscle force; Gas: gastrocnemius muscle force; TP: tibialis posterior muscle force; Gmi: gluteus minimus muscle force; Gma: gluteus maximus muscle force; GS: gemellus superior muscle force; OE: obturator externus muscle force; OI: obturator internus muscle force; Pla: plantaris muscle force; GRF: vertical ground reaction force.

The change patterns of the muscle forces that significantly differ at P1, V0, P2, or in range are shown in Figure 4.

For the V0 point, the soleus muscle force and gastrocnemius muscle force were significantly lower at heel heights of 15 mm and 45 mm compared with a heel height of 0 mm. There were significantly lower tibialis posterior (TP) and plantaris muscle forces at a heel height of 15 mm compared with a heel height of 0 mm. The plantaris muscle force at a heel height of 45 mm was significantly lower compared with a heel height of 0 mm. The obturator externus (OE) and gluteus maximus (Gmax) muscle forces were significantly lower at a heel height of 45 mm compared with a heel height of 15 mm. For point P1, the gluteus minimus (Gmin) muscle force was significantly higher at a heel height of 30 mm compared with a heel height of 15 m. For the P2 point, the gemellus superior (GS) and obturator internus (OI) muscle forces were significantly lower at a heel height of 15 mm compared with a heel height of 0 mm.

### 3.2. Joint Angle

**Figure 5:**
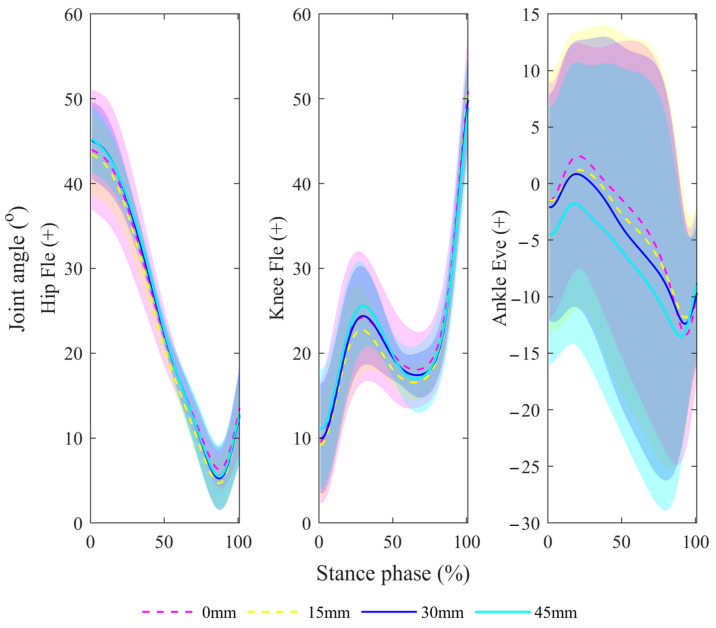
Comparisons of lower-limb joint angles during the stance phase under different shoe conditions. Hip Fle (+): hip flexion (+); Knee Fle (+): knee flexion (+); Ankle Eve (+): ankle eversion (+).

The ankle eversion angle showed the most significant variations. For the joint change range, the hip flexion angle was greater with a heel height of 45 mm compared with a heel height of 15 mm, while the knee flexion angle was lower with a heel height of 45 mm compared with a heel height of 15 mm. For V0, the ankle inversion angle was greater with a heel height of 30 mm compared with a heel height of 0 m. For P1, the ankle was everted when the heel height was 15 mm and the ankle was inverted when the heel height was 45 mm, and the ankle inversion was greater with a heel height of 45 mm compared with a heel height of 30 m. For P2, ankle inversion was greater with a heel height of 45 mm compared with a heel height of 30 mm. More detailed information is shown in Figure 5.

### 3.3. Joint Torque

**Figure 6:**
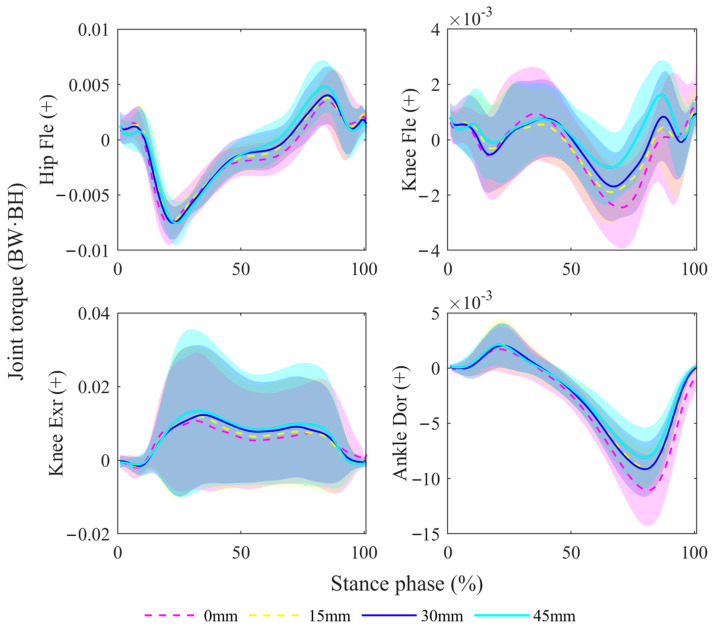
Comparisons of lower-limb joint torque during the stance phase under different shoe conditions. Hip Fle (+): hip flexion torque (+); Knee Fle (+): knee flexion torque (+); Knee Exr (+): knee external rotation torque (+); Ankle Dor (+): ankle dorsiflexion torque (+).

For V0, the hip extension torque was lower at a heel height of 30 mm compared with a heel height of 0 mm. For P2, the knee flexion torque was lower at a heel height of 15 mm compared with a heel height of 0 mm. The ankle plantarflexion torque was lower at a heel height of 15 mm compared with a heel height of 0 mm and was lower at a heel height of 45 mm compared with a heel height of 30 mm. For the range, the ankle dorsiflexion was lower at a heel height of 45 mm compared with a heel height of 0 mm. The knee external rotation torque was lower at a heel height of 45 mm compared with a heel height of 0 mm, and lower at a heel height of 45 mm compared with a heel height of 15 m. More detailed information is shown in Figure 6.

### 3.4. Joint Contact Forces and GRF

**Figure 7:**
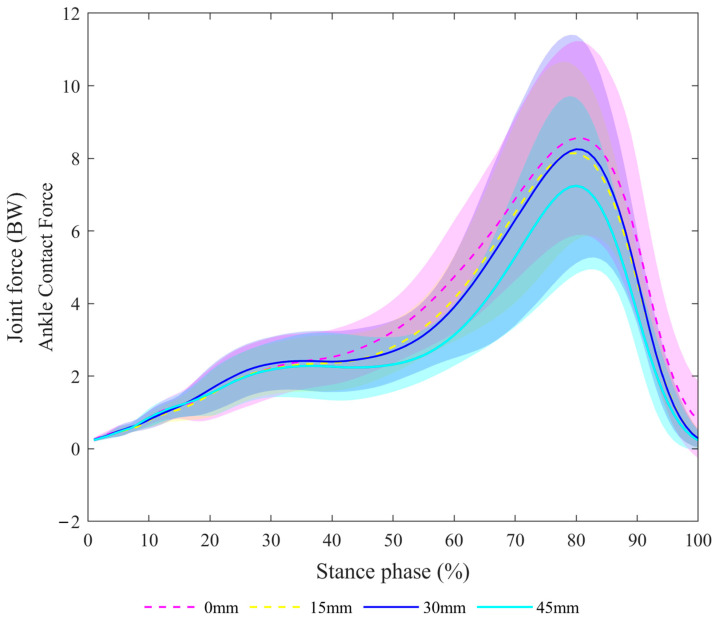
Ankle joint contact force.

For V0, the ankle joint force was lower at a heel height of 45 mm compared with a heel height of 0 mm. For P2, the ankle joint contact force was lower at a heel height of 45 mm compared with a heel height of 15 m. For the range, the ankle joint contact force was lower at a heel height of 45 mm compared with a heel height of 15 mm. More detailed information is shown in Figure 7.

## 4. Discussion

Pregnancy-induced physical changes can alter gait patterns and cause discomfort or pain, and there are limited treatment options for third-trimester musculoskeletal issues. A suitable heel height, one of the most critical parameters of footwear, can help relieve these issues by improving gait stability and correcting biomechanical abnormalities. In this study, a musculoskeletal model was employed to investigate the biomechanical variations in the lower extremities of pregnant individuals across four heel heights. A heel height of 15 mm performed best among the selected heel heights.

A slightly elevated heel height (less than 45 mm) could reduce the muscle force of the soleus and gastrocnemius, as the slightly increased heel height benefits the plantar flexion during propulsion, and the primary function of the calf is to provide plantar flexion [32]. Compared with non-pregnant women and pregnant women in other trimesters, pregnant women in the third trimester have greater ankle plantar flexor torques [33]. The greater ankle plantar flexion torques may induce fatigue and overuse of the soleus and gastrocnemius [34], increasing the risk of muscle strain and leg cramps [35,36], leading to discomfort or pain around the calf region [37]. A slightly elevated heel height (15 mm) also significantly reduces TP muscle force. Situated deep within the posterior region of the lower limb, TP plays a crucial role in stabilizing the medial longitudinal arch and facilitating ankle inversion [38]. Pregnant women are prone to having a lower medial foot arch [39], which can induce higher mechanical demands on the TP muscle [40]. Excessive utilization of the TP muscle influences the coupling patterns between the shank and foot [41], resulting in fatigue or overuse of the TP for pregnant women. Overall, compared with flat shoes, shoes with a slightly elevated heel height are more suitable for pregnant women having issues associated with the posterior muscles of the shank during walking.

A slightly elevated heel height (15 mm) significantly increases the muscle forces of the gluteus maximus and gluteus minimus. The gluteus maximus participates in hip extension and assists in the stability of the pelvis [42,43]. At the same time, pregnant females in the third pregnancy trimester tend to have lower average gluteus maximus muscle activation during the stance phase [33]. The gluteus minimus is active during hip abduction, playing a significant role in pelvic stability during pregnancy [44]. The gemellus superior, obturator externus, and obturator internus participate in the hip joint’s external rotation and play an essential role in maintaining pelvic stability [45,46,47]. The increase in muscle force of these muscles can benefit the stability of the pelvis and help the mass translation of the two lower limbs, which can reduce the risk of lower back and hip pain [48,49,50]. In summary, compared with flat shoes, shoes with a low heel height (15 mm) are more suitable for pregnant women having issues associated with lower back pain during gait.

Elevated heel height significantly increases ankle inversion during the loading response and mid-stance phases, which can increase the risk of ankle inversion sprain [51,52,53]. Elevated heel height influences joint torque more significantly in the ankle than in the hip and knee joints. One possible reason is that pregnant females tend to have increased utilization of the ankle joint, and the ankle joint strategy takes precedence over the hip joint strategy in maintaining balance during pregnancy [12,54]. Elevated heel height reduces the ankle joint contact force, which indicates a lower muscle force of the TP [55], and the decline in TP force can impair the stabilization of the medial longitudinal arch, resulting in easier foot fatigue. In general, a lower heel height (less than 15 mm) is more suitable for pregnant women having ankle inversion sprain.

A slightly elevated heel height is the optimal choice for gravida during walking. This result is consistent with a previous study on walking, which demonstrated that a heel height over 45 mm or less than 5 mm reduced balance [9]. The reduced muscle forces of the soleus and gastrocnemius with a slightly elevated heel height (less than 45 mm) are consistent with previous studies that showed slightly lifting the heel (within 50 mm) can reduce the risk of plantar fasciitis, posterior heel pain, Achilles tendon overuse injuries, or calf cramp [56,57,58]. Although a slightly elevated heel height has been used to reduce or prevent musculoskeletal-related issues like lower back pain, the potential mechanical effects on the muscles still lacked comprehensive investigation [59], and this study filled this gap. As gait analysis was used for the analysis of the biomechanics for pregnant women, the kinematics and kinetics data were used to assess the foundational aspects of the musculoskeletal system of pregnant women, and the results of this analysis, which include the muscle force, joint angle, joint contact force, and joint torque, enhance the detection of musculoskeletal-related problems and the exploration of treatment. Future research should concentrate on two primary areas: (1) conducting gait analysis on pregnant women experiencing musculoskeletal problems under varying heel height conditions and (2) determining which heel heights offer the most benefits for specific musculoskeletal issues. These additional findings could facilitate optimizing heel height selection based on individual musculoskeletal concerns. This study represents the initial exploration of the effects of shoes with different heel heights on pregnant women’s lower-limb gait biomechanics during their third trimester. We comprehensively considered the effects of the four selected heel heights on lower-limb muscle forces, joint contact force, joint angles, and joint torques. We also give specific recommendations on different heel heights based on the musculoskeletal issues that may occur during pregnancy and, overall, a heel height of 15 mm is the best of all heights.

It is essential to acknowledge some inherent limitations. This study only recruited five pregnant females. The findings in this study should be verified using a larger sample size. This study used the individual-preferred walking speed, and the effects of different walking speeds were not investigated. This study focused on the height of the heel, while the shape of the heel is also a factor that influences gait patterns. A larger sample size, different walking speeds and heel shapes, and the height of the arch should be considered in future studies.

## 5. Conclusions

In conclusion, it is more appropriate for pregnant women with issues related to the posterior muscles of the shank to wear shoes with a small heel height (15–45 mm). A heel height (not higher than 15 mm) is more suitable for pregnant women with lower back pain and hips that sway from side to side during walking, unexplained deep muscle pain in the buttocks, or who are prone to ankle sprains. Although there is no golden height for all pregnant females with different conditions, the 15-mm heel height might be optimal overall.

## Figures and Tables

**Table 1:** Detailed information about the participants.

	Age (years)	Height (cm)	Weight (kg)	Pregnant Period (weeks)	Abdomen Circumference (cm)
1	27	160	69	34.43	99
2	28	165	57	30.71	91
3	28	158	70	34.57	103
4	35	168	75	36.86	107
5	30	158	59	35	94
Mean ± SD	29.6 ± 3.21	161.8 ± 4.49	66 ± 7.68	34.31 ± 2.24	98.8 ± 6.5

**Table 2:** Selected items that show significance from the statistical results (adjusted *p*-values).

Parameter	0 vs. 15 mm	0 vs. 30 mm	0 vs. 45 mm	15 vs. 30 mm	15 vs. 45 mm	30 vs. 45 mm
Muscle force						
Soleus	V0 (0.003)	-	V0 (0.014)	-	-	-
Gastrocnemius	V0 (0.009)	-	V0 (0.033)	-	-	-
Tibialis posterior	V0 (<0.001)	-	-	-	-	-
Gluteus minimus	-	-	-	P1 (0.041)	-	-
Gluteus maximus	-	-	-	-	V0 (0.015)	-
Gemellus superior	P2 (0.006)	-	-	-	-	-
Obturator externus	-	-	-	-	V0 (0.012)	-
Obturator internus	P2 (0.012)	-	-	-	-	-
Plantaris	V0 (0.020)	-	V0 (0.043)	-	-	-
Joint angle						
Hip flexion	-	-	-	-	R (0.42)	-
Knee flexion	-	-	-	-	R (0.016)	-
Ankle eversion	-	V0 (0.032)	-	-	P1 (0.002)	P1 (0.013) P2 (0.029)
Joint torque						
Hip flexion	-	V0 (0.032)	-	-	-	-
Knee external rotation	-	-	R (0.017)	-	R (0.028)	-
Knee flexion	P2 (0.048)	-	-	-	-	-
Ankle dorsiflexion	-	-	P2 (0.017) R (0.02)	-	-	P2 (0.047)
Joint contact force						
Ankle	-	-	V0 (0.026)	-	P2 (0.041) R 0.035)	-

Note: *p*-values were adjusted using the Bonferroni correction. -: no significance. Abbreviations: vs. = versus; P1 = peak 1; P2 = peak 2; V0 = valley; R = change range.

## Data Availability

The data presented in this study are available on request from the corresponding author. The data are not publicly available due to privacy issues of the participants.
